# Body Space in Social Interactions: A Comparison of Reaching and Comfort Distance in Immersive Virtual Reality

**DOI:** 10.1371/journal.pone.0111511

**Published:** 2014-11-18

**Authors:** Tina Iachini, Yann Coello, Francesca Frassinetti, Gennaro Ruggiero

**Affiliations:** 1 Department of Psychology, Laboratory of Cognitive Science and Immersive Virtual Reality, Second University of Naples, Caserta, Italy; 2 Department of Psychology, Research Unit on Cognitive and Affective Sciences, University of Lille, Lille, France; 3 Department of Psychology, University of Bologna, Bologna, Italy; Max Planck Institute for Human Cognitive and Brain Sciences, Germany

## Abstract

**Background:**

Do peripersonal space for acting on objects and interpersonal space for interacting with con-specifics share common mechanisms and reflect the social valence of stimuli? To answer this question, we investigated whether these spaces refer to a similar or different physical distance.

**Methodology:**

Participants provided *reachability-distance* (for potential action) and *comfort-distance* (for social processing) judgments towards human and non-human virtual stimuli while standing still (passive) or walking toward stimuli (active).

**Principal Findings:**

Comfort-distance was larger than other conditions when participants were passive, but reachability and comfort distances were similar when participants were active. Both spaces were modulated by the social valence of stimuli (reduction with virtual females vs males, expansion with cylinder vs robot) and the gender of participants.

**Conclusions:**

These findings reveal that peripersonal reaching and interpersonal comfort spaces share a common motor nature and are sensitive, at different degrees, to social modulation. Therefore, social processing seems embodied and grounded in the body acting in space.

## Introduction

The space around the body is of fundamental importance to interact with objects and persons. In the literature, two traditions of research have explored body space: one about peripersonal space in the neuro-cognitive field, one about personal space in the social psychology field.

In the neuro-cognitive field, this space is defined in relation to the possibility of acting with objects: ‘peripersonal space’ is the portion within arm reaching distance, whereas ‘extrapersonal space’ is the area outside arm reaching [1]–[5]. Peripersonal space is the first margin between the surface of our body and the external world. For this reason some authors have conceived it as a protective buffer surrounding the body and prompting defensive actions [6]–[8].

Neuro-functional studies have shown that peripersonal space is represented by highly integrated multisensory and motor processes in frontal-parietal and posteromedial areas [4], [9]–[12]. Moreover, peripersonal space seems also sensitive to social-emotional components and social interactions [13]–.

In the neuro-cognitive literature, a well known experimental task to assess the size of peripersonal space is the reachability judgment: participants have to evaluate if visual stimuli presented at various distances from the body are reachable or not [2], [17]. People are quite accurate in estimating the extension of their peripersonal space in relation to the length of their arm [18], [19]. However, reachability judgments are also influenced by environmental properties, emotional state and dangerousness of the situation [2], [17], [20], [21]. For example, the size of peripersonal space reduces when dealing with dangerous objects that may threaten physical integrity [6].

In social psychology, the term ‘personal space’ defines an emotionally tinged zone around the body that people feel like “their private space” and cannot be intruded by others without causing discomfort [15], [22], [23]. The distance individuals maintain between themselves and others can be defined “interpersonal space”. People tend to react to spatial violations by extending distance from intruders when feeling in hostile and uncomfortable situations and, vice-versa, by reducing distance when feeling in friendly and comfortable situations [20], [22]–[24].

In the social psychology literature, a typical task to assess the size of interpersonal space is based on comfort-distance judgments provided through the ‘stop-distance’ paradigm: participants have to stop the interactant at the point where they still feel comfortable with the other’s proximity [21], [23], [25]–[27]. Different kinds of stimuli representing the interactant have been used: real confederates, paper and pencil materials, manikins [28]. Overall, the size of this space may contract or expand depending on situational, emotional and individual characteristics such as gender [23], [25], [29], [30].

The parallel reading of peripersonal and interpersonal space literature suggests that there is an intrinsic relationship between action, social interaction and spatial processing. The use of spatial distance is inherent in action with objects and interaction with other people. In line with Lloyd [14], from an ‘action-centered’ perspective the interpersonal space can be seen as the physical space where some social actions occur on the basis of their emotional and motivational relevance. One can thus question the relationship between peripersonal space for acting on objects and interpersonal space for interacting with con-specifics.

The conceptual definitions and the experimental paradigms used to study peripersonal space stress the sensorimotor aspect of spatial processing, whereas the conceptual definitions and the experimental paradigms used to study interpersonal space stress the social value of spatial processing. For this reason, studies on peripersonal space have mainly focused on the individual-object relationship, whereas studies on interpersonal space focused on the individual-individual relationship. Both literatures agree on the fact that spatial distance is inherent in our actions and social interactions, and that the size of spatial boundaries around the body are revealing of underlying functions and mechanisms. The issue addressed here is whether interpersonal space overlaps with peripersonal space when participants interact with their physical and social environment.

In the present study we explored the relationship between peripersonal space and interpersonal space in the interaction with humans and objects by using the immersive virtual reality (IVR) technology. Once immersed in a virtual room, female and male participants interacted with computer-driven virtual stimuli: young males and females, anthropomorphic robot and cylinder. Participants could stand still while virtual stimuli approached them (passive approach) or could walk toward immobile virtual stimuli (active approach). They had to stop themselves or stop the virtual stimuli in order to provide two types of measures: *reachability-distance,* i.e. distance at which participants thought they could reach the virtual stimuli; and *comfort-distance*, i.e. distance at which participants felt comfortable with the virtual stimuli. These tasks were chosen for two reasons: theoretically, the first one is more sensitive to sensorimotor properties for acting in the here and now, whereas the second one is more sensitive to emotional/social properties for interacting with others; methodologically, the two ways of measuring the spatial behavior are easily comparable.

Finally, the reliability of IVR to study social interactions has been proved in several studies [26], [31].

Our hypothesis was that reachability-distance and comfort-distance share a common aspect that is rooted in the motor nature of the space around the body. Thus from an action-centered perspective [14], these distances should be more similar when we can act towards stimuli (active approach) than when we cannot (passive approach). Indeed, peripersonal reaching space is linked by definition to action; at the same time, approaching-avoidant movements are necessary to define the desired comfort area. Instead, when another person moves toward us, we do not have direct control over the interaction. Therefore, we could be particularly sensitive to possible spatial violations and, as a preparation to defend, we would enlarge our body space. This effect should be more sensitively expressed in comfort than reaching space. Moreover, since it has been recently shown that the size of peripersonal space shrinks in the presence of a person as compared to a manikin [16], we expect a reduction of distances with human as compared to non-human virtual stimuli. Among non-human stimuli, we used an anthropomorphic robot (i.e. a “machine” with a human body-like appearance) and a cylinder (i.e. a geometrical object with no social valence). If body space is finely sensitive to the social valence of stimuli, distances should be smaller with the robot than the cylinder. This pattern, even if more expected for interpersonal space, should also be present in peripersonal space to confirm its sensitivity to social modulation. Finally, the proxemics literature shows that male and female participants differ in their spatial behavior: females tend to expand the space around their body as compared to males since they are more sensitive to intrusions and safety characteristics of contexts [25]. Therefore, we expect a male-female main effect and an interaction between the gender of participants and the virtual stimuli.

### Experiment

#### Ethics Statement

Participants gave written consent to take part in the study. Recruitment and testing were in conformity with the the requirements of the 2008 Helsinki Declaration. The local Ethics Committee of the Department of Psychology, Second University of Naples specifically approved this study.

## Materials and Methods

### Participants

Thirty-six right-handed students (18 women), aged 18–37 years (*M* = 22.3, *SD* = 4.4), education (years, *M* = 15.1, *SD* = 1.7) were recruited from the Second University of Naples (Italy) in exchange for credits to examination. All participants had normal or corrected-to-normal vision. The Edinburgh Handedness Inventory [32] was used to measure the handedness (*mean score* = 90.7, *SD* = 3.2).

### Setting and Immersive Virtual Reality (IVR) equipment

The experiment was carried out in the Laboratory of Cognitive Science and Immersive Virtual Reality, Department of Psychology, Second University of Naples, Caserta (Italy). The IVR equipment is installed in a rectangular room (5 m×4 m×3 m) and includes the 3-D Vizard Virtual Reality Toolkit Devices for Integrated VR Setups and Position Tracking System. Virtual stimuli were presented through the nVisor SX (NVIS, USA) head mounted display (HMD) with two displays providing stereoscopic depth (approximately 30 times a sec.). The stereoscopic images run at 1280×1024 resolution, refreshed at 60 Hz. The virtual scenario spanned 60 degrees horizontally by 38 degrees vertically. Graphics card used Vizard softwares (WorldViz, USA). Head orientation was tracked by a three-axis orientation sensor (InertiaCube3; Intersense, USA) and head position by a passive optical tracking system (Precision Position Tracker, PPT-H4; WorldViz, USA). Graphics displayed in the HMD were updated on the basis of sensed position and orientation of participant’s head. Moreover, the Data Glove, a glove equipped with 14 tactile-pressures sensors providing the sense of hand movement, was also used. Graphics modeling were created by 3D Google Sketch Up 7.0 free-software. The position and orientation tracking systems allowed participants to realistically experience dynamic and stereoscopic visuo-motor input as if they were in front of natural stimuli.

### Virtual Stimuli

#### Virtual environment

The virtual room measured 3 m×2.4 m×3 m. It consisted of green walls, white ceiling and grey floor. On the floor, a dashed white line from the participants’ starting position until the end of the virtual room was traced. Participants could move forward/backward without colliding with any real obstacle [26].

#### Virtual agents

**Figure 1:**
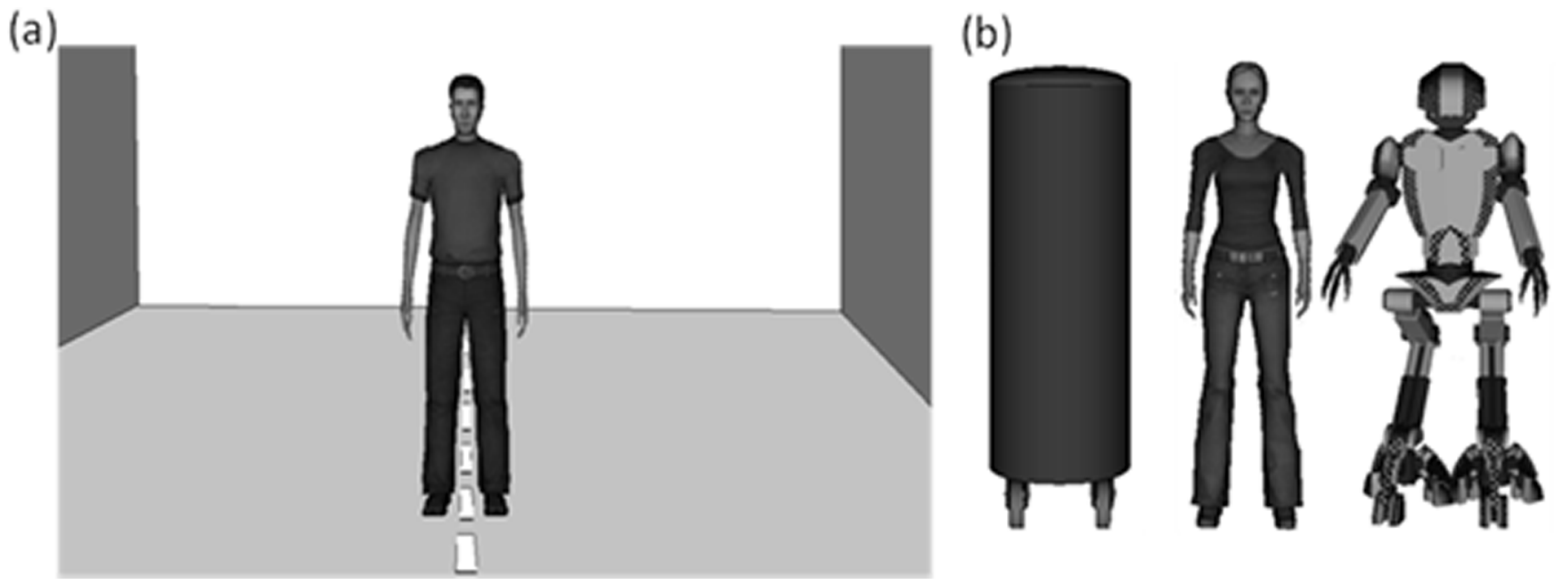
Virtual stimuli and environment. Panel (**a**) shows participant’s perspective when a virtual agent (e.g., an adult male) frontally appeared. A straight dashed white line placed on the floor traced the path that participants and virtual agents followed during both approach-conditions. Panel (**b**) shows (from the left) the other virtual stimuli used: a cylinder, an adult woman, and an antrophomorphic-robot.

Pilot studies were performed to select the avatars most similar to human beings (rated on a 5-point scale). The selected human avatars represented male and female adults aged about 30 years and perceived as representation of Italian citizens. As shown in Figure 1, male and female avatars kept their arms extended along the body. An anthropomorphic robot and a cylinder were also used (see Figure 1). The height of the virtual stimuli was 175 cm. The gaze of human avatars and anthropomorphic robot was kept looking straight ahead throughout the experimental sessions and their facial expression was neutral [26]. Since distance can be influenced by familiar size in impoverished visual environments [33], in a control experiment 20 participants (half females) judged the height of each virtual stimulus while positioned at three counterbalanced positions from them (1.5 m, 2 m and 3 m). The results showed that all virtual stimuli were perceived with the same height independently of the distance from the perceiver (F<1).

### Procedure

The experimenter introduced participants to the IVR devices to make them acquainted with the virtual world. All participants received written instructions about the task that were then orally repeated by the experimenter. Then, there was a familiarization phase. Participants wore the HMD and the Data Glove and were allowed to explore freely the virtual room. Data Glove was used to make participants perceive their arm fully stretched in the virtual scene. Through the HMD, participants were fully immersed in the virtual room where they could see the virtual stimuli and could make extensive exploratory movements. They could not see any part of the physical world. During this familiarization phase, participants were asked to describe their perception of the virtual environment and their interaction with the humans avatars and objects. Participants reported they had the feeling of being like “inside a movie”, “in a realistic world”, and “with realistic persons”. Nobody claimed problems with the IVR devices or with virtual room and stimuli. After the familiarization session, participants were led by the experimenter on a pre-marked starting position and had to hold a joystick in their dominant right hand. Throughout the experimental session, the participants stood with their arms extended along their body, similarly to the posture assumed by virtual humans (see Figure 1).

The experimental session was divided in four blocks corresponding to the experimental conditions: (i) passive-comfort distance, (ii) active-comfort distance; (iii) passive-reachability, (iv) active-reachability. For each block, participant received a training session in which an example of the entire procedure was shown. Each block started with a short presentation of the instructions (2 s) followed by a fixation cross (300 ms). Afterwards, the testing phase started. In half of the trials participants provided *comfort-distance* judgments (instruction: “press the button as soon as the distance between yourself and the virtual stimulus makes yourself feel uncomfortable”), in the other half they provided *reachability-distance* judgments (instruction: “press the button as soon as you can reach with your hand the virtual stimulus”). This procedure was repeated in passive and active approach conditions. In the “passive approach”, participants stood still and saw virtual stimulus walking towards them at a constant speed (0.5 m.s^−1^) until they stopped them by pressing the button. In the “active approach”, the virtual stimuli remained motionless and participants walked towards them (0.5 m.s^−1^) until they stopped and at the same time pressed the button. In both conditions the path between participants and stimuli was 3 m long. Walking movements of human avatars reproduced the natural swing of biological motion. After pressing the button, the virtual stimulus disappeared and participants had to return to their starting position. Once there, the experimenter pressed a key that prompted the subsequent trial. Participants walked forwards and backwards by following the white dashed-line on the virtual ground. The experimenter supervised and helped participants when necessary. A 5 min break was introduced every two blocks with the HMD taken off. Each virtual stimulus was presented 4 times within each block for a total of 64 trials. Order of blocks was counterbalanced across participants according to a Latin square design. Within each block, order of trials presentation was quasi-randomized. Each block lasted about 6 min. At the ending of each block there was a manipulation check: participants had to report which task they were instructed to perform. In the post-experimental final interview, participants were asked if they were aware of the purpose of the experiment and nobody claimed so. Moreover, to explore participants’ feelings when immersed in the virtual world with virtual stimuli, participants were asked to indicate which approach condition and which virtual stimulus they found pleasant or not. Participants reported they preferred the active rather than the passive condition. The majority of female participants reported they had no particular preference but disliked particularly the virtual male and the cylinder. The majority of male participants indicated they found particularly pleasant their experience with virtual females but not with virtual males. At the ending, the experimenter measured the length (cm) of participants’ dominant arm from the acromion to the extremity of the middle finger.

### Data analysis

We measured the distance at which the participants stopped themselves or the virtual stimuli according to the task (Reachability or Comfort distance) and the condition (Active or Passive). The IVR system tracked the participants’ position at a rate of approximately 18 Hz. The computer recorded participant’s position in the virtual room by continuously computing the distance between the marker placed on participants’ HMD and virtual stimuli. In each condition, this tracking system allowed to record the participant-virtual stimulus distance (in cm). Participant’s arm length was then subtracted from the mean distance. Within each block and for each type of stimulus the mean participant-virtual stimulus distance was then computed.

The mean distances obtained in the different experimental conditions were compared through a four-way ANOVA including participants’ Gender as between-participant factor and Distance (Reachability-Comfort distance), Approach (Passive-Active approach), and Virtual stimuli (male, female, cylinder, robot) as within-participant factor. Bonferroni post-hoc test was used to analyze significant effects. The magnitude of the effect sizes was expressed by partial eta squared (*η^2^_p_*).

## Results

Statistical analysis revealed a significant effect of Gender (F(1, 34) = 11.250, p<0.002, *η^2^_p_* = 0.25), due to overall distance from virtual stimuli being larger in females (M = 58.02 cm, SD = 36.43 cm ) than males (M = 36.58 cm, SD = 29.84 cm). The variable Distance was not significant (F(1, 34) = 1.926, p = 0.17: Reachability-distance = 43.57 cm, SD = 30.49; Comfort-distance = 51.03 cm, SD = 39.71). A main effect of the variable Approach emerged (F(1, 34) = 36.525, p<0.0001, *η^2^_p_* = 0.52), with participants keeping a larger distance in Passive (M = 61.20 cm, SD = 45.18 cm) than Active (M = 33.40 cm, SD = 25.02 cm) condition. A main effect of Virtual stimuli appeared (F(3, 102) = 27.903, p<0.001, *η^2^_p_* = 0.45). Post-hoc analysis showed that participants kept a larger distance from the cylinder (64.55 cm) than other stimuli (male = 45.15 cm, female = 35.80 cm, robot = 46.09 cm, all ps <0.001), and a smaller distance from virtual females than other stimuli (all ps <0.05). No difference was found between virtual robot and male (p = 1).

**Figure 2:**
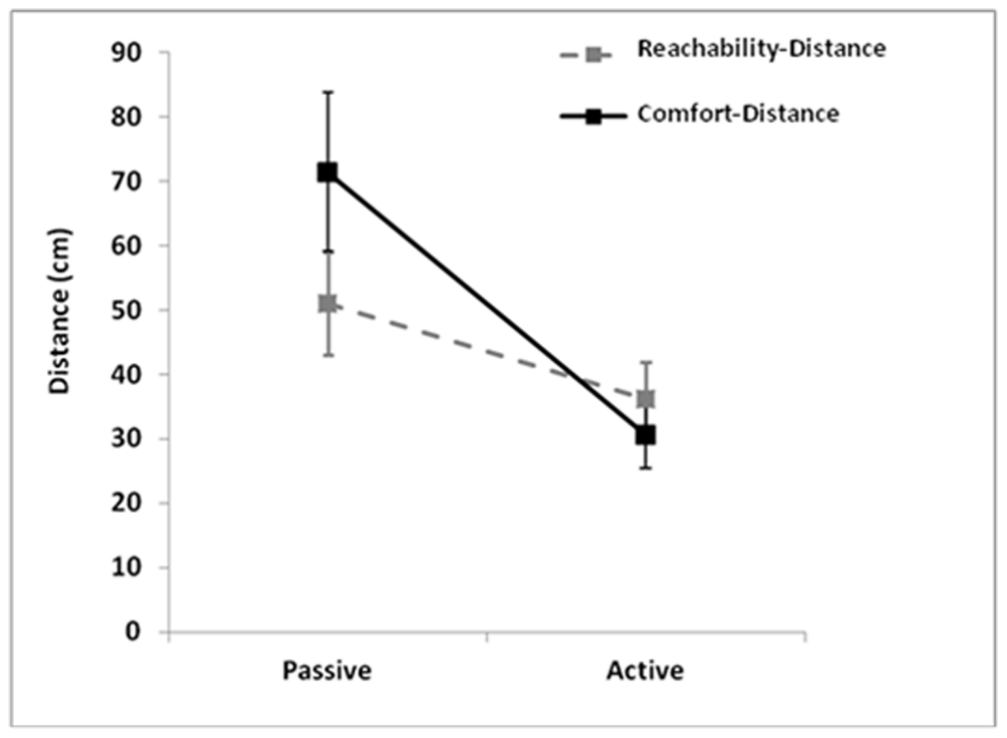
Interaction distance-approach condition. Mean (cm) reachability-distance and comfort-distance as a function of passive/active approach-conditions.

The ANOVA showed a significant Distance × Approach interaction: (F(1, 34) = 11.916, p<0.001, *η^2^_p_* = 0.26, see Figure 2). Reachability-distance was larger in the Passive than Active approach (p<0.05). Comfort-distance was also larger in the Passive than Active approach (p<0.001). However, in the Passive approach, Comfort-distance was significantly larger than Reachability-distance (p<0.005), whereas in the Active approach no difference was found between Comfort and Reachability distances (p = 1).

**Figure 3:**
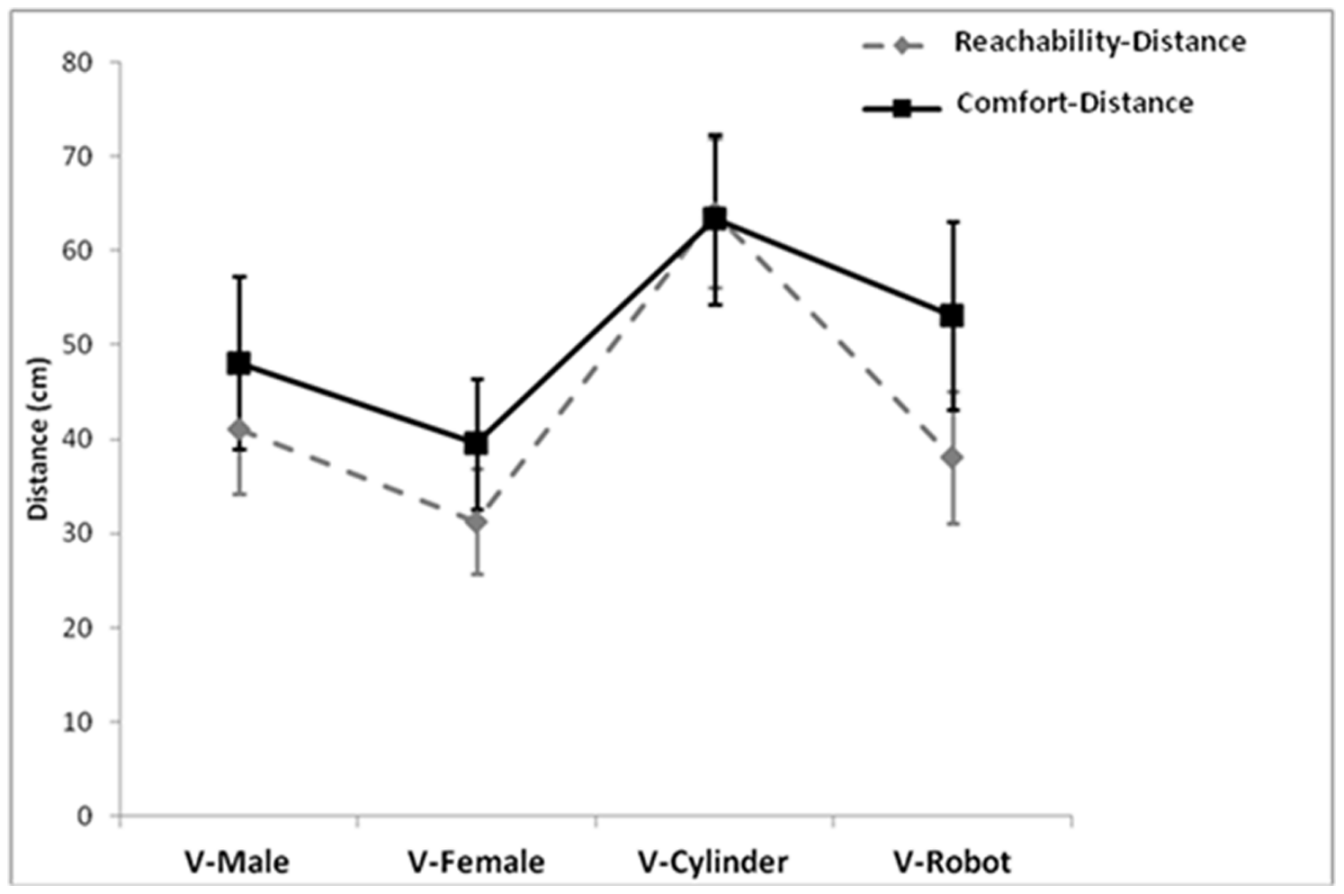
Interaction distance-virtual stimuli. Mean (cm) reachability-distance and comfort-distance as a function of the interaction with virtual stimuli.

The Virtual stimuli factor interacted with Distance: (F(3, 102) = 3.411, p<0.05, *η^2^_p_* = 0.09). As shown in Figure 3, when comparing Reachability- and Comfort-distances in function of the virtual stimuli, only one difference emerged: in presence of the robot Comfort-distance was larger than Reachability-distance (p<0.001). Moreover, Comfort-distance was reduced when dealing with virtual females than robot (p<0.005). Instead, in presence of the cylinder Reachability and Comfort distances almost overlapped and were larger than with other stimuli (at least p<0.002; Comfort-distance with robot approached significance, p = 0.07).

**Figure 4:**
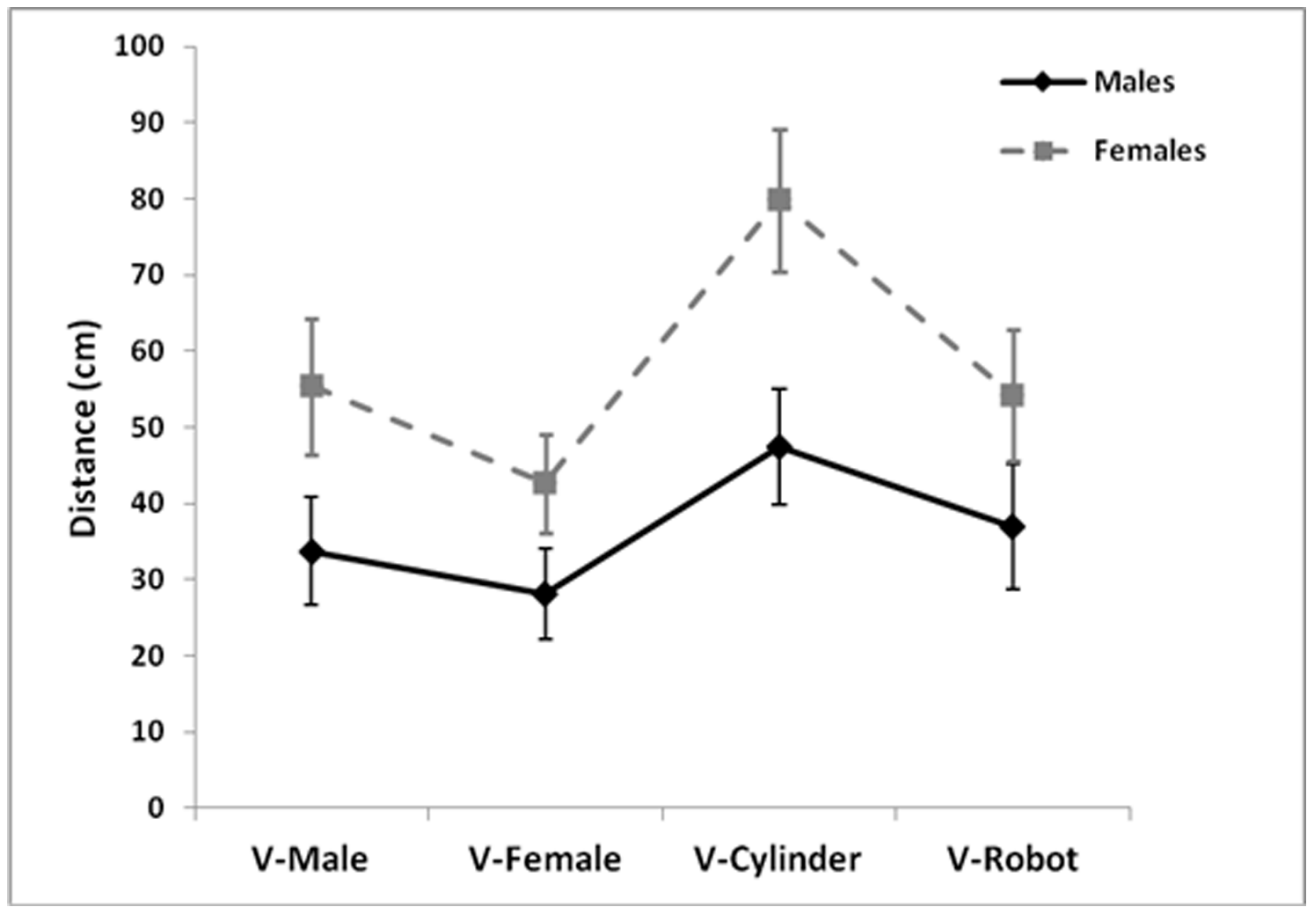
Interaction participants’ gender-virtual stimuli. Mean distance (cm) of male and female participants as a function of the interaction with virtual stimuli.

Participants’ gender affected the spatial behavior with Virtual stimuli: (F(3, 102) = 3.053, p<0.05, *η^2^_p_* = 0.08, see Figure 4). Female participants kept a larger distance from cylinder than other stimuli and than males dealing with all stimuli, at least p<0.001). Instead, male participants reduced space in presence of virtual females as compared to cylinder (p<0.001) and to female participants dealing with virtual males (p<0.01). When comparing the two groups, no difference between male-male and female-female dyads emerged (p = 1).

Finally, to exclude that the variation of only one distance (reachability or comfort) could be sufficient to explain the whole pattern of data, we separately analyzed Reachability and Comfort distances by means of a 2 (Gender) × 2 (Passive/Active Approach) × 4 (Virtual stimuli) mixed ANOVA.

As regards Reachability-distance, significant main effects of Gender (F(1, 34) = 5.997, p<0.05, *η^2^_p_* = 0.15 with females>males) and of Approach condition (F(1, 34) = 20.424, p<0.001, *η^2^_p_* = 0.37 with Passive>Active) were found. Finally, distance varied as a function of the type of stimulus (F(3, 102) = 27.385, p<0.0001, *η^2^_p_* = 0.45). Bonferroni post hoc test showed that distance from cylinder was larger than all other stimuli, distance from virtual females was shorter than males (all ps <0.01).

The same effects were replicated with Comfort-distance: significant main effects of Gender (F(1, 34) = 7.28, p<0.05, *η^2^_p_* = 0.18, with females>males), Approach condition (F(1, 34) = 27.841, p<0.001, *η^2^_p_* = 0.45, with Passive>Active) and Virtual stimuli (F(3, 102) = 11.337, p<0.0001, *η^2^_p_* = 0.25). Regarding the last effect, distance was larger from cylinder than males and females, and shorter from females than robot (all ps <0.01).

Therefore, the splitted ANOVAS showed that both Reachability−/Comfort-distances were affected by the same factors (gender of participants, approach conditions, type of virtual stimuli).

## Discussion

What is the relationship between sensorimotor spatial processes and social processes in the modulation of the space around the body? To answer this question, this study assessed whether the size of the portion of space that people judged reachable and comfortable was similar or different, and whether judgments are influenced by the active or passive way of interacting with the environment. Although few studies have suggested that peripersonal space, similarly to interpersonal space, can reflect social components [13], [16], these two spaces have never been compared to assess to what extent they share common aspects.

The results showed that, considering the different approaches, the two distances were similar in some aspects and different in others. More specifically, a difference emerged in the passive-approach since comfort distance was larger than reachability distance, whereas in the active approach no difference was found. As also shown by separate analyses, both reachability and comfort distances were larger in the passive than active condition, but the effect was particularly strong with comfort distance. Since in the passive condition participants were approached by others, notably unfamiliar others, the larger comfort than reachability distance in this case could reflect an increased need of controlling the interaction and maintaining a feeling of safety.

Participants in the passive condition preferred a larger comfort than reachability distance, suggesting that in a social interactive situation which is not under the control of ones’ own action, comfort perception is associated with maintaining others at larger distances. This may be associated with the specific safety value of interpersonal space, which is widely influenced by the emotional characteristics of approaching and/or threatening stimuli [2], [6]. When an intruder invades our body space, there is an activation of the amygdala in response to this violation [20]. People tend to compensate unwanted intimacy by expanding their body space and preparing to avoid a collision with the intruder [2], [20], [22], [23]. Moreover, in the passive condition it could be more difficult to anticipate others’ behavior, particularly with virtual stimuli whose movement patterns can be unnatural (objects) or not fully constrained by biological laws (humans) [34]. By contrast, when participants could actively move, reachable and comfort distances were controlled on the basis of their fully predictable behavior. Although in both conditions participants could decide when stopping the movement, only in the active condition they were controlling their throughout behavior. The finding that reachability and comfort distances have a similar size in the active approach, that is when participants can act with stimuli, may suggest that the motor component of the task influenced both distance judgments in the same way. In other words, it is possible that motor predictive processes subtending reachability judgments [2], also contribute to specifying comfortable social distance [14].

The other finding which suggests a communality between the two spaces is that both are modulated by human vs non-human stimuli. As expected, their size was expanded with virtual objects and reduced with virtual humans. This pattern is consistent with data showing a smaller peripersonal space with a human confederate than a manikin and confirms that also this space reflects a social component [16]. Both reachability and comfort distances around the body seem endowed with finely tuned mechanisms for processing social information and reflect gender-related effects.

Indeed, the distance from virtual stimuli is reduced with virtual females as compared to males and enlarged with cylinder as compared to robot. As discussed below, the shorter distance from virtual females could reflect attraction and self-protection mechanisms [25], [35]–[37]. The fact that body space was smaller with the robot might be due to its anthropomorphic appearance that evoked a human-like interaction [38]. Instead, the cylinder cannot be perceived as the “subject” of a social interaction and, interestingly, in that case reachable and comfort space had the same size. However, in presence of the robot comfort-distance was larger than reachable-distance. The robot is a special case: it is an object but with the appearance of a human body. Therefore, participants behaved with the robot as if it were a male and this behavior was reflected in the peripersonal size. But the robot is not human and this ambiguity can be disturbing: this is reflected in the enlargement of interpersonal space. This suggests that peripersonal and interpersonal spaces show a different sensibility for the stimuli with or without social connotation.

In line with previous virtual reality studies where participants walked towards and around virtual agents, the results showed that female participants maintained a larger distance from virtual agents as compared to their male counterparts [26]. The gender effects reported in the social literature are often interpreted as a consequence of arousal regulation and the necessity to ensure a stable self-protection. According to the Equilibrium Theory proposed by Argyle and Dean [36], each social interaction involves approach and avoidance behaviors that provoke the optimal regulation of personal distance. When a situation involves stranger interactants, females exhibit a more defensive behavior than males and this is expressed in an enlargement of their personal space [25], [35], [39].

Gender also affected the spatial behavior with virtual stimuli. Women enlarged body distance when dealing with the cylinder, i.e. the object with no social valence, as compared to other stimuli. This might be interpreted as a consequence of their sensitivity for the possibility of communicating and the social meaning of a situation [21], [22], [39]. Men reduced body distance from virtual females. Finally, women treated similarly virtual male/female humans and robot. Instead, Takayama and Pantofaru [38] found that females expanded space more than males in presence of a real robot and interpreted the effect as due to women’ lower tolerance for frontal interaction. Clearly, the different spatial behavior among sexes may reflect socialization differences rather than biological differences [25], [30].

The usage of IVR technology deserves a last comment. From a methodological perspective, the IVR system has the advantage of ensuring a complete control over the variables of interest (virtual humans’ appearance and behavior, environmental context) while maintaining a good level of ecological validity and realism [31], [40], [41]. This is important since previous research has typically used observational methods and real humans as confederate at risk of losing experimental control. However, further research is needed to clarify limitations and vantages of virtual reality.

From a theoretical perspective, the results bring on the issue of social presence, that is the degree to which new interactive media are able to prompt a human-like interaction [31]. It is important to note that participants in our experiment reported they felt as if they were in a realistic context and evaluated differently the virtual stimuli: for example, the majority of men reported they liked more the virtual female than other stimuli, whereas women reported they liked less the virtual male and cylinder than other stimuli. The different spatial behavior with virtual objects and humans, and the gender effects would suggest a high degree of “humanization” and sense of self-presence in the virtual world. To what extent these new virtual media may change our minds and social interactions is a matter of further research.

In conclusion, spatial behavior is a key aspect of our socio-emotional life and possibility of acting. We act not only with objects to satisfy our needs and wishes, but also with people to express the emotions we feel and the desired quality of our interaction. The crucial factor modulating the size of personal space, then, could be represented by approach-avoidance actions that reduce or increase this size depending on the social-emotional valence of external stimuli and by motor plans to react to rewarding or threatening objects [7], [8], [14], [20]. While the interpersonal comfort space stresses the first factor and could pre-alert about potential spatial violations, the peripersonal action space stresses the second factor and seems more sensitive to the immediate action context. This leads to conclude that there is more a quantitative than a qualitative difference between them.

Our findings thus highlight a close relationship between basic visuomotor-spatial processing and complex social processing. They are consistent with embodied approaches of perception and cognition which argue that processing sensory information, whether in an individual or social context, is influenced by the body and the action system [42]–[44]. In line with this theoretical framework, the present findings suggest that social distance is influenced by the experience of the body acting in space, suggesting that bodily states and simulation of information in the brain’s modality-specific systems for perception, action, and introspection support social processing [45].
